# Simulation-Driven Spatial Frequency Domain Imaging and Deep Learning for Subsurface Fruit Bruise Discrimination

**DOI:** 10.3390/foods15081397

**Published:** 2026-04-17

**Authors:** Jinchen Han, Yanlin Song, Xiaping Fu

**Affiliations:** School of Information Science and Engineering, Zhejiang Sci-Tech University, Hangzhou 310018, China; 2023210701003@mails.zstu.edu.cn (J.H.); 2025210701007@mails.zstu.edu.cn (Y.S.)

**Keywords:** spatial frequency domain imaging, fruit bruise discrimination, data simulation, optical property inversion, surface profile correction

## Abstract

Conventional spatial frequency domain imaging (SFDI) based optical property inversion is inefficient, while deep learning methods suffer from heavy reliance on large-scale real datasets. To address this contradiction, a simulation-driven approach for subsurface fruit bruise discrimination was proposed. An SFDI simulation environment was built with Blender to generate 800 paired datasets of diffuse reflectance images and optical transport coefficients, overcoming the high cost and long cycle of real dataset acquisition. We designed the CBAM-GAN-U-Net model and adopted surface profile correction in the prediction method to eliminate curved surface-induced non-planar distortion, with the whole method validated on liquid phantoms, green apples and crown pears. This prediction method achieved high accuracy in predicting the reduced scattering coefficient *μ_s_*′, with NMAE of 0.021 ± 0.007 (phantoms), 0.039 ± 0.012 (severely bruised green apples) and 0.044 ± 0.015 (severely bruised crown pears), outperforming U-Net and GANPOP. Based on the predicted *μ_s_*′, a discrimination strategy combining coefficient of variation, mean ratio and receiver operating characteristic (ROC) curve analysis was adopted, attaining 100% accuracy for non-bruised/bruised fruit discrimination, with misclassification rates of 6% (green apples) and 8% (crown pears) for mild/severe bruise differentiation. This method enables accurate subsurface fruit bruise detection, providing a reliable technical solution for the fruit and vegetable industry and helping reduce postharvest supply chain losses.

## 1. Introduction

Fruits undergo the entire supply chain process, from field harvesting to terminal marketing. They are inevitably subjected to mechanical stresses such as compression and impact. These stresses can lead to tissue damage in fruits. In the early stage, this damage often appears as subsurface bruising. The flesh tissue is damaged in this case, while no visible marks show up on the peel. As a result, the external appearance of bruised fruits is almost the same as that of sound fruits. This makes it extremely difficult to identify bruised fruits reliably through conventional visual inspection. With prolonged storage, the damaged tissue keeps deteriorating and spreading. It not only causes internal decay of the affected fruit. It also may lead to contact-based cross-contamination in entire boxes or batches of produce. This results in substantial economic losses along the fruit and vegetable supply chain [1]. Therefore, developing efficient and accurate techniques for subsurface bruise detection is of great practical importance. These techniques can help reduce postharvest losses and ensure quality stability throughout the supply chain.

In the field of nondestructive fruit bruise detection, mainstream technologies include Near-Infrared Spectroscopy (NIRS), Raman Spectroscopy (RS), Hyperspectral Imaging (HSI), and Fluorescence Spectroscopy (FS), all with their own advantages and inherent limitations in practical use. Spectroscopy-based techniques only provide single-point measurement results without complete two-dimensional spatial information. They also have poor model generalizability across fruit varieties, and cannot offer reliable quantitative support for subsequent automated image analysis and bruise identification. HSI delivers high accuracy for early bruise detection by capturing sample spectral and spatial information at the same time, but it is limited by long data acquisition cycles, redundant data processing and high equipment costs. It cannot meet the needs of real-time detection and efficient image analysis. More critically, none of the above methods can effectively separate the absorption and scattering properties of light in biological tissues, which makes it difficult to accurately identify subsurface occult bruising from the mechanistic view of tissue microstructural changes. In contrast, Spatial-Frequency Domain Imaging (SFDI) can effectively overcome these core limitations and provides a reliable technical solution for high-precision nondestructive fruit bruise detection.

SFDI is a non-invasive optical technique for turbid media. It combines operational simplicity with quantitative capability [2,3]. This technique has been widely applied to analyze optical transport coefficients and assess the quality of fruits like apples [4,5] and pears [6,7]. It is also used for other related quality evaluations [8]. SFDI uses sinusoidally modulated structured illumination. It acquires reflected images of the sample surface at multiple phase shifts. We can extract the medium’s key optical transport coefficients quantitatively through demodulation and optical parameter inversion. These coefficients are the absorption coefficient *μ_a_* and the reduced scattering coefficient *μ_s_*′. Previous studies have shown that optical transport coefficients obtained by SFDI are sensitive to tissue structural changes induced by fruit bruising. This is especially true for the reduced scattering coefficient. These coefficients enable effective identification of subsurface bruises [9] and quantitative assessment of bruise severity [10]. These findings provide a reliable optical characterization foundation for the nondestructive detection of subsurface fruit bruises.

SFDI has demonstrated distinct advantages in fruit optical inspection. However, it has a major limitation. Optical property inversion during data processing is very time-consuming. This leads to a relatively low overall detection efficiency. To solve this problem, many studies have explored improvement strategies. Among these strategies, integrating SFDI with machine learning and deep learning techniques has become a key research focus. Early research focused on conventional machine learning approaches. For example, random forest regression (RFR) was used to accelerate SFDI-based optical property inversion [11]. Artificial neural networks (ANN) were also applied to directly estimate the absorption coefficient and reduced scattering coefficient from spatial frequency domain images [12]. With the rapid development of deep learning, more advanced network architectures have been introduced into this field. Deep residual networks (DRN) were used to establish mappings between diffuse reflectance images and optical parameters [13]. Long short-term memory networks (LSTM) were leveraged to improve the accuracy of temporally dependent inversions [14]. To further improve both efficiency and accuracy at the same time, researchers have proposed several new methods. These include single-snapshot SFDI combined with conditional generative adversarial networks (cGAN) [15], hybrid frameworks integrating GANs with multilayer perceptrons (MLP) [16], and lightweight architectures such as dual U-Net models [17] and ultra-compact deep neural networks [18]. These methods significantly reduce computational time while maintaining high prediction accuracy. They bypass traditional data processing steps and effectively enhance computational efficiency. Thus, these integrated approaches have become a major trend in recent SFDI research.

The aforementioned SFDI–deep learning hybrid approaches have notable efficiency advantages. However, they also introduce new limitations. Training deep learning models requires large-scale, high-precision paired input–label datasets. These datasets must be obtained through multiple labor-intensive stages. The stages include real fruit sample preparation, SFDI system data acquisition, and conventional high-accuracy optical property inversion. This dataset acquisition process has three main drawbacks: long acquisition cycles, high costs and limited sample availability. It substantially increases the research barrier. It also contradicts the original objective of improving SFDI detection efficiency.

Traditional SFDI inversion suffers from extremely low computational efficiency, while large-scale paired training datasets required for deep learning-enabled SFDI optimization are scarce, labor-intensive, and costly to acquire. To resolve this fundamental conflict, this study aims to establish a simulation-dataset-driven technical framework for SFDI-based optical property analysis and subsurface fruit bruise detection. The core and ultimate goal of this work is to achieve rapid, accurate, and non-destructive subsurface bruise discrimination on real fruit samples, so as to mitigate postharvest losses in the fruit supply chain.

To this end, this study will first construct a physically realistic SFDI simulation environment to generate low-cost, high-quality labeled simulation samples, which will be exclusively used to support the training of deep learning models. We will then develop an improved CBAM-GAN-U-Net model to realize rapid and accurate prediction of optical transport coefficients in fruit tissues. Finally, we will conduct systematic, multi-dimensional validation of the model’s performance and generalizability on completely independent real sample sets, including liquid phantoms, green apples, and crown pears, to establish a full technical framework for subsurface fruit bruise discrimination.

## 2. Materials and Methods

### 2.1. SFDI System and Method

**Figure 1:**
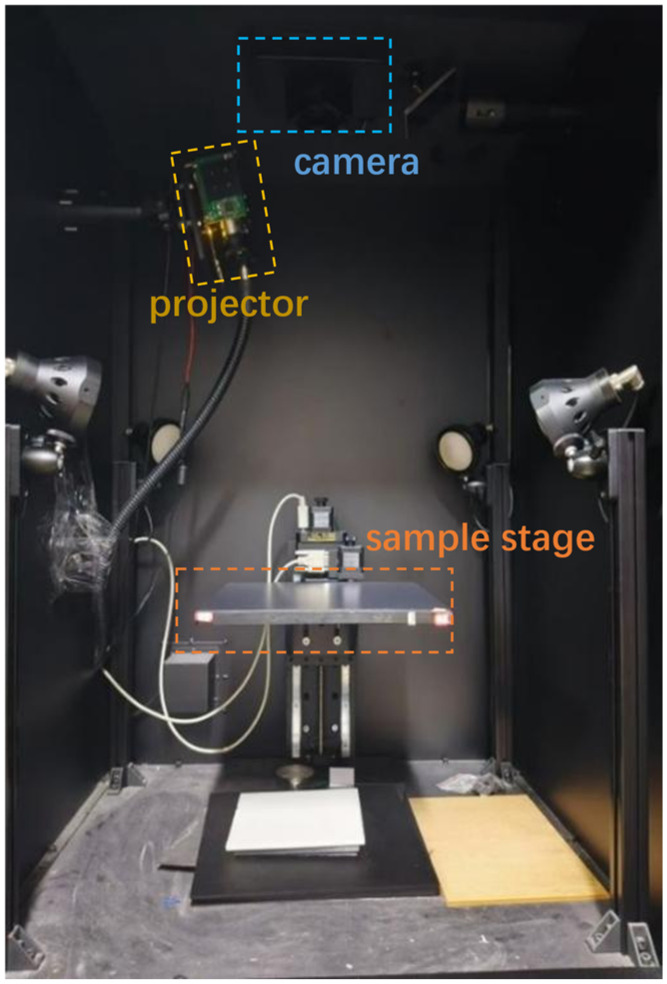
SFDI system.

We used an SFDI system in this study (Figure 1). Its core imaging component is a GaiaField-V10E-AZ4 hyperspectral camera (Dualix Spectral Imaging, Chengdu, China). This camera has a spectral range of 400–1000 nm and a spectral resolution of 2.8 nm. Its 16-bit output ensures a wide dynamic range for the acquired signals. The camera is equipped with an integrated translational push-broom scanning mechanism. This mechanism enables stable image acquisition and ensures the accuracy of frequency-domain distribution imaging.

#### 2.1.1. SFDI System

The system’s projection module includes a series of key components. It has a halogen light source and a light source power controller (both from Ocean Electro-optics, Changchun, China), an optical fiber (Dualix Spectral Imaging, Chengdu, China) and a digital light projector (DLi CEL5500, Texas Instruments, Austin, TX, USA). It also has a linear polarizer mounted at the front of the projection lens (Beijing Optical Century Instrument, Beijing, China). All these components work together to realize a stable projection of sinusoidal structured illumination. They also enable effective control of polarization characteristics.

The system is fitted with a dedicated sample stage. It is constructed with an electric translation stage and stepper motors (both from Beijing Optical Century Instrument, Beijing, China). This sample stage allows precise positioning and orientation adjustment of the samples. Two accompanying software programs are used for the experimental operation. SpecView is applied for imaging parameter configuration and data acquisition, which ensures experimental controllability. SpecSight is used for subsequent data processing and analysis, including image demodulation and optical parameter inversion.

#### 2.1.2. Principle of SFDI

In this study, SFDI is employed to obtain the optical transport coefficients of fruits, with the primary ones being the absorption coefficient *μ_a_* and the reduced scattering coefficient *μ_s_*′. The fundamental principle [19] is based on illuminating the target sample with sinusoidally modulated structured light and acquiring the diffusely reflected intensity signals at three different phase shifts (0, 2π/3, and 4π/3). The diffuse reflectance amplitude envelope is then extracted by demodulating the three phase-shifted images. The simplified demodulation formula is given in Equation (1):

(1) $$M_{AC}(x,f_{x})=\frac{\sqrt{2}}{3}{\left\{{\left[I_{1}-I_{2}\right]}^{2}+{\left[I_{2}-I_{3}\right]}^{2}+{\left[I_{3}-I_{1}\right]}^{2}\right\}}^{\frac{1}{2}}$$

where $I_{1}$, $I_{2}$, and $I_{3}$ correspond to the light intensity images acquired at the three phase shifts.

The amplitude envelope is calibrated using a standard reflectance reference with a reflectivity of 0.99 to obtain the quantitative diffuse reflectance $R_{d}(f_{x})$. Subsequently, the optical transport coefficients are retrieved through nonlinear least-squares fitting based on a forward model, as expressed in Equation (2):

(2) $$R_{d}(f_{x})=\frac{3A\mu_{s}^{\prime }/\mu_{tr}}{\left(\frac{\mu_{eff}^{\prime }}{\mu_{tr}}+1)(\frac{\mu_{eff}^{\prime }}{\mu_{tr}}+3A\right)}$$

where $\mu_{tr}=\mu_{a}+\mu_{s}^{\prime }$ is the transport coefficient, $\mu_{eff}^{\prime }={\left(3\mu_{a}\mu_{tr}+K\right)}^{\frac{1}{2}}$, K=2πf. Proportionality constant $A=\frac{1-R_{eff}}{2(1-R_{eff})}$, $R_{eff}=0.0636n+0.668+\frac{0.71}{n}-\frac{1.44}{n^{2}}$ is the refractive index of the sample.

We aimed to detect subsurface fruit bruises in this study. We experimentally evaluated candidate wavelengths across the full spectral range. We ultimately selected 720 nm as a suitable imaging wavelength for the research. At shorter wavelengths, diffuse reflectance is strongly affected by scattering from fruit surface lenticels and peel textures. This leads to low image brightness. It also makes background noise mask the optical contrast between bruised and sound fruit regions. At longer wavelengths, the fruit peel shows excessively high reflectance. This easily causes image overexposure and intensity signal saturation. It further hinders the reliable extraction of quantitative optical parameters. 720 nm falls in the mid-spectral region. This wavelength effectively avoids scattering interference and insufficient brightness at low wavelengths. It also evades the overexposure risk at high wavelengths. Thus, it achieves a favorable balance among different competing spectral effects. We set the spatial frequency to 0.2 mm^−1^ in the experiments. This setting mitigates the insufficient sensitivity to subsurface bruises caused by low spatial frequencies. It also ensures the stable extraction of bruise-related features. In this way, it effectively improves the overall detection accuracy.

Notably, this single-wavelength and single-spatial-frequency design is optimized for the rapid detection of subcutaneous fruit bruising, which supports the real-time performance of the proposed method. Meanwhile, we acknowledge the limitation of this simplification. The current parameter combination is optimized for the tested apple and pear samples, and its robustness for other fruit varieties with different peel characteristics or bruise depths needs further verification.

### 2.2. Simulation-Driven Dataset Generation

A simulation dataset was constructed for training the optical transport coefficient prediction model of fruit bruising. The SFDI process was simulated for this dataset with Blender, an open-source 3D rendering software developed by the Blender Foundation. Blender is an open-source 3D rendering platform equipped with the Cycles ray-tracing renderer. The Henyey–Greenstein phase function is employed by this renderer to model internal light scattering within objects. This phase function is also widely used in Monte Carlo simulations of light transport in biological tissues [20]. Optical transport coefficients of biological tissues have been successfully simulated with Blender in previous studies [21,22].

**Figure 2:**
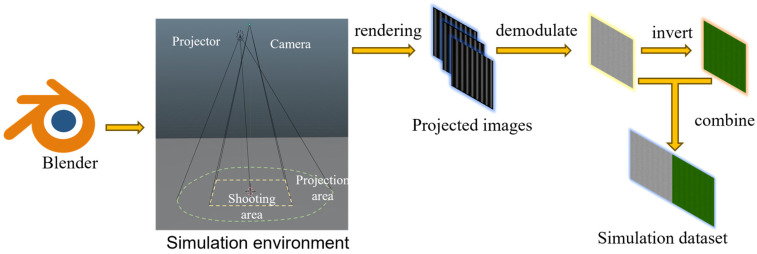
Workflow of Blender-based SFDI simulation dataset generation.

In this work, the simulation environment was first constructed in Blender to replicate the configuration of the actual SFDI system. Simulation data were then generated through rendering, followed by data collection and processing to obtain training samples, as illustrated in Figure 2.

#### 2.2.1. Model Construction

The application scenarios of SFDI in biological tissue imaging and the planar imaging characteristics of fruit samples were taken into account. A planar simulation model was thus constructed in Blender. The planar sample was defined as a square surface with dimensions of 100 mm × 100 mm. Scattering and absorption properties were customized via Blender’s material editor. Specifically, a combination of a transparent bidirectional scattering distribution function (BSDF) and a subsurface scattering shader was employed for light scattering simulation. Light absorption was modeled with a transparent BSDF combined with a refractive BSDF, whose refractive index was set to 1.43. The relative weights of the scattering and absorption components were adjusted. Variations in the optical transport properties of different fruit tissues were thus reproduced. The Cycles ray-tracing engine was adopted for the simulation, with the light source power set to 3.5 W. This setting ensured the physical realism of the light transport process. The image resolution was uniformly set to 256 × 256 pixels to achieve a balance between simulation accuracy and data processing efficiency.

#### 2.2.2. Spatial Frequency Projection and Raw Data Acquisition

The demodulation requirements of SFDI were met in the simulation. Two types of spatial frequency images were projected onto the planar model via the projection system in Blender. A total of four raw images were yielded in this process. One image was set with a spatial frequency of 0 mm^−1^. The other three images were set with a spatial frequency of 0.2 mm^−1^, with different phase shifts (0, 2π/3, and 4π/3). The camera viewpoint in Blender was fixed perpendicular to the planar surface. This setting ensured precise alignment between the projected patterns and the imaging region. Frequency distortion caused by viewing angle deviations was thus avoided. Parameter configuration was implemented successively. The raw images were automatically rendered and saved after each round of parameter configuration.

#### 2.2.3. Data Processing

The four raw images acquired in Blender were processed with standard SFDI demodulation and inversion procedures. Diffuse reflectance images of the simulation dataset were obtained through demodulation. Corresponding optical transport property maps were retrieved through inversion. These maps include the absorption coefficient *μ_a_* and the reduced scattering coefficient *μ_s_′.* Both coefficient maps were normalized to the range of [0, 1]. They were then encoded as 8-bit RGB images for use as labels. In these images, the R channel corresponds to *μ_a_*, the G channel corresponds to *μ_s_*′, and the B channel is fixed at zero. Paired data were generated through this entire procedure. The paired data consist of diffuse reflectance images (inputs) and RGB optical property maps (labels).

#### 2.2.4. Dataset Generation

Batch generation of training data was realized via Blender’s keyframe animation technique. The variation ranges and step sizes of the optical transport coefficients (scattering and absorption coefficients) were defined as keyframes in the Blender animation editor. This setting was designed to cover the optical property range of fruit tissues. The animation frame range was specified for the simulation. Blender’s material optical parameters were automatically adjusted at each frame. Four raw images were rendered accordingly for each frame setting. Paired datasets were generated through batch demodulation and inversion processing. These datasets consisted of diffuse reflectance images and RGB optical property maps. A total of 800 sets of simulated data were acquired in the simulation. These simulated data were divided into a training set and a validation set at a 7:3 ratio for model training. The training set contained 560 samples, and the validation set included 240 samples.

### 2.3. Deep Learning–Based Prediction of Optical Transport Coefficients

A deep learning prediction model was developed for this study. It targets the mapping between SFDI measurements and optical transport properties. The model is constructed based on a generative adversarial network (GAN) framework. Its baseline architecture is derived from the generative adversarial network prediction of optical properties (GANPOP) model, as reported in related studies [23]. Key limitations exist in the baseline model for subsurface fruit bruise detection. These limitations include insufficient capture of weak local features and relatively large prediction errors at bruise boundary regions. A convolutional block attention module (CBAM) was thus introduced for task-specific enhancement. The proposed CBAM-GAN-U-Net model was developed from this architectural improvement. In addition, two comparative models (GANPOP and U-Net) were implemented for subsequent performance evaluation.

**Figure 3:**
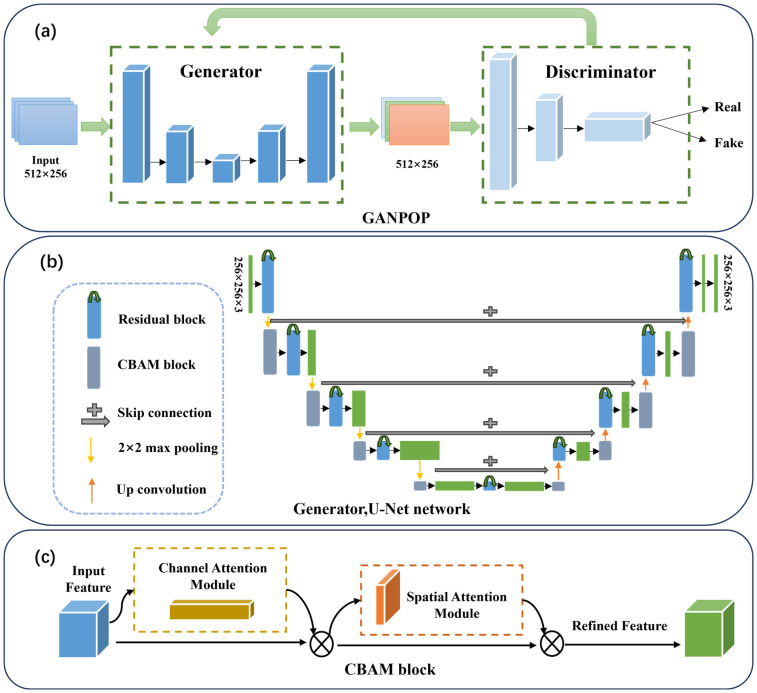
Schematic diagram of the GAN-U-Net-Attention model: (**a**) GANPOP model; (**b**) Generator, U-Net network; (**c**) CBAM block.

A generator–discriminator adversarial training framework is adopted by the model (Figure 3a). A 256 × 256 diffuse reflectance image and its corresponding 256 × 256 three-channel optical transport coefficient label image are concatenated along the width dimension. A 512 × 256 input image is formed through this concatenation. During the training process, the left 256 × 256 half of the input image is used as the generator input. The right 256 × 256 half is employed as the ground-truth label for loss computation [24].

In the GANPOP model, the generator is a U-Net architecture integrated with residual blocks (Figure 3b). It is composed of an encoder, a decoder, and skip connections [23]. Cross-layer skip connections are employed in the generator. Fine-grained details are thus preserved. An optical transport property prediction map is output by the generator, with the same spatial resolution as the label image. A three-layer fully convolutional classifier is adopted by the discriminator. Real and generated sample pairs are distinguished by this classifier. Adversarial feedback is also provided to the generator by the discriminator. Based on this baseline architecture, a CBAM module is embedded into the proposed model’s generator. The module is placed after each down-sampling stage and before each up-sampling stage (Figure 3c). Dual-dimensional optimization is implemented in the proposed model to enhance feature extraction. Feature channels strongly correlated with optical transport properties are selected via channel attention. Local weak-feature regions associated with bruises are focused on through spatial attention. Through this dual-dimensional optimization, the baseline network’s limited sensitivity to mild bruises and bruise boundary features is effectively overcome by the proposed model.

A sequential channel-attention–spatial-attention structure is adopted by the CBAM module [25]. Global average pooling and max pooling are employed by the channel attention submodule to estimate the contribution of each feature channel. Channel-wise weights are learned through a fully connected network. Redundant channels dominated by background noise are thus suppressed effectively. Spatial feature statistics are captured by the spatial attention submodule to localize bruise-related local feature peaks. The contribution of pixels within bruise regions is enhanced accordingly. The insufficient focus of the baseline model on weak local features is also compensated for by this spatial attention mechanism.

The loss function adopts a composite strategy combining GAN adversarial loss and L1 loss, and the Adam optimizer is used for training. The total loss function is defined as:

(3) $$L_{total}(G,D)=L_{GAN}(G,D)+\lambda \cdot L_{1}(G)$$

where λ is the regularization coefficient, set to 60 in this study. $L_{GAN}(G,D)$ denotes the adversarial loss, expressed as:

(4) $$L_{GAN}(G,D)=E_{x,y\sim pdata}[\log D(x,y)]+E_{x\sim pdata}[\log(1-D(x,G(x)))]$$

in which, G represents the generator with embedded CBAM modules, D denotes the discriminator, x is the diffuse reflectance image, and *y* is the ground-truth optical transport coefficient image.

The pixel-wise loss $L_{1}(G)$ is defined as:

(5) $$L_{1}(G)=E_{x,y\sim pdata}[∥y-G(x)∥]$$

### 2.4. Evaluation Metrics

To objectively quantify the prediction accuracy of deep learning models for optical transport coefficients, this study adopts the normalized mean absolute error (NMAE), peak signal-to-noise ratio (PSNR), and structural similarity index (SSIM) as the primary evaluation metrics. These indicators provide a comprehensive assessment from three perspectives: absolute error magnitude, pixel-level distortion, and structural similarity between the predicted and ground-truth images.

The NMAE eliminates dimensional effects and quantifies the average absolute deviation between predicted values and ground-truth values. A smaller NMAE indicates higher prediction accuracy. It is defined as:

(6) $$NMAE=\frac{\sum_{i=1}^{T}\left|p_{i}-p_{i,ref}\right|}{\sum_{i=1}^{T}p_{i,ref}}$$

where $p_{i}$ is the predicted pixel value, $p_{i,ref}$ is the ground-truth (label) pixel value, and *T* denotes the total number of pixels.

To verify whether the performance differences between the proposed CBAM-GAN-U-Net model and the baseline models (U-Net, GANPOP) are statistically significant, a two-tailed paired Student’s t-test was adopted for pairwise model comparison.

All statistical tests were performed based on the NMAE results of the test samples, with a pre-set significance level of α = 0.05. The significance criteria were defined as follows: *p* < 0.05 was considered a statistically significant difference, *p* < 0.01 was considered an extremely significant difference, and *p* < 0.001 was considered an ultra-significant difference.

The PSNR measures the ratio between image signal power and noise power. A higher PSNR indicates lower pixel-level distortion and better image quality. It is expressed as:

(7) $$PSNR=10\mathrm{lg}\frac{255^{2}}{MSE}$$

where MSE is the mean squared error between the predicted image and the ground-truth image. The value 255 represents the maximum gray-level value after coefficient normalization, and is mapped to the range 0–255.

The SSIM evaluates image similarity from three aspects—luminance, contrast, and structure. A value closer to 1 indicates stronger structural consistency. SSIM is defined as:

(8) $$SSIM=\frac{\left(2\mu_{\overset{⌢}{p}}\mu_{p}+C_{1})(2\sigma_{\overset{⌢}{p}p}+C_{2}\right)}{\left(\mu_{\overset{⌢}{p}}^{2}+\mu_{p}^{2}+C_{1})(\sigma_{\overset{⌢}{p}}^{2}+\sigma_{p}^{2}+C_{2}\right)}$$

where $\mu_{\overset{⌢}{p}}$ and $\mu_{p}$ are the mean values of the predicted image and the ground-truth image, respectively, $\sigma_{\overset{⌢}{p}}$ and $\sigma_{p}$ are their standard deviations, $\sigma_{\overset{⌢}{p}p}$ denotes the covariance between them. $C_{1}={\left(0.01\times 255\right)}^{2}$,$C_{2}={\left(0.03\times 255\right)}^{2}$.

A two-tailed paired Student’s t-test was used to verify the statistical significance of performance differences between the proposed model and baseline models (U-Net, GANPOP). All tests were performed on paired data from the same set of test samples, with a significance level set to α = 0.05. *p* < 0.05 was defined as a statistically significant difference, *p* < 0.01 as extremely significant, and *p* < 0.001 as ultra-significant.

### 2.5. Experimental Materials

#### 2.5.1. Fruit Sample Preparation

Green apples and crown pears with pronounced differences in surface characteristics were selected. The generalizability of the proposed method was verified using these two fruit types. The peel of green apples shows a uniform green color with a smooth texture. Prominent lenticel structures are absent on its surface, resulting in a relatively homogeneous surface structure. In contrast, the peel of crown pears is generally light yellow. Visible lenticels and sporadic brown spots are present on its surface, leading to a heterogeneous surface structure. All fruit samples were purchased from a local fruit market. The samples were stored in a controlled laboratory environment for 24 h prior to experimentation. This storage step was implemented to minimize the influence of initial environmental differences on the physiological state of the samples. Visual inspection was employed during the sample selection stage. Fruits with congenital defects or pre-existing mechanical damage were excluded. Only samples without visible surface bruising were selected for subsequent grouping and preparation.

Green apple samples were numbered from A001 to A100, and crown pear samples were numbered from P001 to P100. For both fruit types, a total of 100 independent samples were prepared with a balanced grouping design for the two core detection tasks of this study.

The first task is a non-bruised/bruised binary classification task. 50 non-bruised healthy samples and 50 artificially bruised samples were prepared for each fruit type. For green apples, non-bruised samples correspond to A001–A050, and bruised samples correspond to A051–A100; for crown pears, non-bruised samples correspond to P001–P050, and bruised samples correspond to P051–P100.

The second task is a mild/severe bruise severity grading task. Among the 50 bruised samples of each fruit type, 25 mildly bruised samples and 25 severely bruised samples were prepared. For green apples, mild bruise samples correspond to A051–A075, and severe bruise samples correspond to A076–A100; for crown pears, mild bruise samples correspond to P051–P075, and severe bruise samples correspond to P076–P100.

Standardized subsurface bruises were artificially induced via a custom-designed pendulum impact device. The core component of the device is a metal sphere with a mass of 0.031 kg, fixed to one end of a nylon string, with the other end of the string connected to a pivot structure, forming a pendulum system capable of free oscillation about the pivot. For the impact experiment, the pendulum was lifted to a position parallel to the horizontal plane and then released, allowing the metal sphere to swing downward along an arc and strike the equatorial region of the fruit sample. Notably, each fruit sample received only one single standardized impact in this experiment, with each sample corresponding to one independent intact fruit, which strictly avoids the risk of pseudo-replication in all subsequent statistical analysis.

Different bruise severities were quantitatively generated by adjusting the length of the nylon string. Mild bruising corresponds to an impact energy of 0.05 J (string length is 0.161 m). Severe bruising corresponds to an impact energy of 0.10 J (string length is 0.322 m).

In total, 200 independent fruit samples were included in this study, comprising 100 green apples and 100 crown pears. The optical transport coefficients of all samples were measured via conventional high-precision SFDI technology, and the established real fruit dataset was used as a completely independent test set to validate the detection performance of our model trained exclusively on the simulation-generated dataset.

#### 2.5.2. Liquid Phantom Preparation

The prediction accuracy of the deep learning model for fruit optical transport coefficients was validated. A total of 30 liquid phantom samples were prepared for this validation experiment. The samples were with controllable absorption coefficients *μ_a_* and reduced scattering coefficients *μ_s_*′. The optical transport properties of the phantoms were designed to match the actual optical parameter ranges of apples and pears.

The coefficient ranges of the liquid phantoms were determined based on experimentally measured fruit optical properties reported in the literature. Experimentally measured optical property ranges of apples and pears have been documented in previous studies [8,26,27,28,29]. For these two fruit types, the absorption coefficient *μ_a_* typically ranges from 0 to 0.4 mm^−1^, and the reduced scattering coefficient *μ_s_*′ ranges from 0 to 3 mm^−1^. Optical parameter fluctuations induced by fruit bruising were taken into consideration. The requirement for sufficient adjustment margins during subsequent experiments was also considered. The coefficient ranges of the liquid phantoms were thus extended for both optical coefficients. The absorption coefficient *μ_a_* range was extended to 0–0.5 mm^−1^, and the reduced scattering coefficient *μ_s_*′ range was extended to 0–4 mm^−1^. Thereby, the extended parameter ranges fully cover the optical transport characteristics of both normal and bruised fruit tissues.

Deionized water was used as the base material for the liquid phantoms. It features high optical transparency and a stable composition, which contributes to the reduction in background optical interference. Specific reagents were added to control the optical properties of the phantoms. India ink (Royal Talens, Apeldoorn, The Netherlands) was used as the absorber to precisely adjust the absorption coefficient *μ_a_*. Titanium dioxide (TiO_2_) powder (model T104946-100 g, Aladdin Reagent Co., Shanghai, China) was employed as the scattering agent. The TiO_2_ particles possess good dispersibility. This property enables stable and reproducible control of the reduced scattering coefficient *μ_s_*′ in the liquid phantoms.

### 2.6. Fruit Surface Profile Correction Method

The simulation dataset of this study was generated based on planar models, while real fruits possess non-planar curved surface geometries. In practical operation, it is necessary to use the fruit surface profile correction method to correct fruit data.

We used planar models for simulation to strictly match this classical physical model, so that the model can learn the essential mapping relationship between diffuse reflectance and optical transport coefficients, rather than fitting the geometric distortion features caused by irregular curved surfaces. This design ensures the physical authenticity of the simulation dataset and avoids the model learning invalid interference features unrelated to optical properties. However, the non-planar curved surface of real fruits will cause significant geometric distortion in the SFDI-collected diffuse reflectance images, which is manifested as the typical “center-bright and edge-dark” intensity distribution. This distortion will introduce systematic errors into the optical property prediction and break the input consistency between real sample data and the planar simulation dataset. Therefore, surface profile correction is a necessary preprocessing step for SFDI applied to irregular curved samples, which has been widely verified and applied in the field of fruit quality detection [4,7].

For the above reasons, a validated four-step phase-shifting structured light method was employed for surface profile correction in this study [30]. Four sinusoidal fringe patterns with fixed phase shifts are generated as the core procedure. These patterns are sequentially projected onto the fruit surface by a projector. Deformed fringe images are synchronously captured, and the absolute phase is subsequently retrieved. Three-dimensional height information of the fruit surface is extracted from the absolute phase. This three-dimensional height information is then applied to correct the original diffuse reflectance data. System errors caused by non-planar surface geometries are thus eliminated, and input consistency between simulation data and real fruit data is ensured.

The profile correction is defined as:

(9) $$R_{correct}=R_{uncorrect}/c$$

where $R_{uncorrect}$ is the original (uncorrected) diffuse reflectance value of the sample, $R_{correct}$ is the corrected diffuse reflectance value, and c is the correction coefficient, defined as the ratio of the height at pixel (x,y) to the maximum height of the fruit surface.

The diffuse reflectance data obtained after demodulation contain errors induced by the non-planar surface structure. Applying contour correction to the demodulated diffuse reflectance data effectively removes the systematic errors caused by surface geometry. In practical fruit optical transport property prediction tasks, all diffuse reflectance inputs to the model were subjected to surface profile correction to improve prediction accuracy.

### 2.7. Fruit Bruise Discrimination Method

Previous studies and our experimental validation have confirmed that the reduced scattering coefficient *μ_s_*′ is highly sensitive to the microstructural changes in fruit pulp tissue induced by mechanical bruising, while the absorption coefficient *μ_a_* exhibits no statistically significant difference between non-bruised and bruised tissues. Accordingly, this study takes *μ_s_*′ as the core optical indicator for bruise characterization, and establishes a two-step sequential discrimination framework to achieve accurate binary classification of fruit bruise states and quantitative grading of bruise severity.

The first step is binary discrimination between non-bruised and bruised samples. The coefficient of variation (CV) of the global *μ_s_*′ distribution across the whole fruit sample is adopted as the core discriminative indicator. Classify by a threshold. Samples with CV values above the threshold are classified as bruised, while those below the threshold are classified as non-bruised.

The second step is severity grading of mild and severe bruises for samples identified as bruised in the first step. The mean ratio of *μ_s_*′ between the bruised region and the adjacent sound tissue region of the same sample is used as the core grading indicator. To address the potential distribution overlap of mean ratio values between mild and severe bruise groups, receiver operating characteristic (ROC) curve analysis is adopted for threshold optimization. Bruised samples with mean ratio values below the threshold are classified as severe bruise, while those above the threshold are classified as mild bruise.

The CV is defined as the ratio of the standard deviation to the mean and is used to quantify the dispersion of the *μ_s_*′ distribution within a fruit sample. This metric effectively distinguishes between non-bruised and bruised states. In sound fruits, the tissue structure is relatively uniform, resulting in a stable *μ_s_*′ distribution and a low CV value. In contrast, bruised samples exhibit pronounced differences in *μ_s_*′ between damaged and sound regions, leading to increased distribution dispersion and significantly elevated CV values. The CV is calculated as:

(10) $$CV=\frac{\sigma (\mu_{s}^{\prime })}{{\bar{\mu }}_{s}^{\prime }}$$

where $\sigma (\mu_{s}^{\prime })$ denotes the global standard deviation of the reduced scattering coefficient data, and ${\bar{\mu }}_{s}^{\prime }$ represents the global mean value of the reduced scattering coefficient.

This metric is computed based on the global data of the entire *μ_s_*′ map, enabling effective discrimination without the need to extract specific local regions. This approach avoids potential sample bias that may arise from local region selection.

The mean ratio is defined as the ratio between the mean *μ_s_*′ value of the bruised sample and that of sound samples of the same fruit type, and is used to quantify bruise severity. Mild bruising causes limited cellular damage and results in a relatively small decrease in *μ_s_′,* whereas severe bruising leads to extensive cell rupture and a pronounced reduction in *μ_s_*′, yielding a substantially lower mean ratio. The mean ratio is calculated as:

(11) $$M=\frac{{\bar{\mu }}_{s,D}^{\prime }}{{\bar{\mu }}_{s,N}^{\prime }}$$

where M denotes the mean ratio, ${\bar{\mu }}_{s,D}^{\prime }$ is the mean reduced scattering coefficient of the damaged region, and ${\bar{\mu }}_{s,N}^{\prime }$ is the mean reduced scattering coefficient of the sound region.

Partial overlap exists in the mean ratio distributions of mildly bruised and severely bruised samples. ROC curve analysis was thus introduced to optimize the bruise discrimination threshold. The ROC curve characterizes the relationship between the true positive rate (TPR, namely sensitivity) and the false positive rate (FPR) under different classification thresholds. The false positive rate is defined as FPR = 1 − specificity. This curve provides an intuitive evaluation of the trade-off between the correct identification of positive samples and the exclusion of negative samples. A curve positioned closer to the upper-left corner indicates superior classification performance [31,32].

The area under the curve (AUC) is employed as a quantitative metric for evaluating ROC performance, with values ranging from 0.5 to 1. A larger AUC value indicates a stronger overall discriminative capability of the model. An AUC value of 0.5 corresponds to random guessing, while an AUC value of 1 represents perfect classification. The optimal classification threshold is determined by maximizing the Youden index, with the calculation formula defined as: Youden Index = Sensitivity + Specificity − 1. This criterion achieves a balance between sensitivity and specificity, and optimal overall performance is thus yielded for mild/severe bruise classification.

## 3. Results and Discussion

The proposed CBAM-GAN-U-Net model was trained on 800 groups of Blender-generated paired SFDI simulation datasets. All experiments were validated on completely independent real sample sets (30 liquid phantoms, 100 green apples, 100 crown pears), following a “simulation pre-training, real sample testing” paradigm. Real sample SFDI images were corrected for surface profile, input into the model to predict optical properties, then used for bruise discrimination via CV and mean ratio strategy, with results compared to the ground-truth bruise state for accuracy quantification, to verify the effectiveness and generalization of the proposed method.

### 3.1. Evaluation of Fruit Surface Contour Correction

The simulation dataset of this study was generated with planar models, whereas real fruits possess non-planar surface geometries. This induces geometric distortion in the diffuse reflectance data acquired by SFDI, which further impairs the accuracy of optical transport coefficient prediction and fruit bruise discrimination. Therefore, the effectiveness of the contour correction technique was evaluated through both CV–based quantitative analysis and visual inspection of image features.

**Figure 4:**
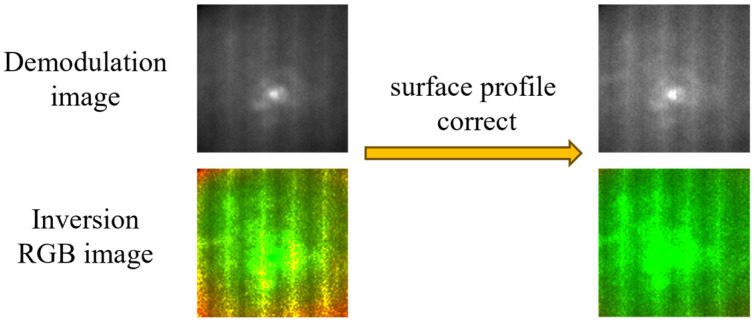
Comparison of fruit demodulation images and inversion RGB images before and after fruit surface profile correction.

As presented in Figure 4, uncorrected diffuse reflectance images exhibit a pronounced center-bright and edge-dark characteristic. This phenomenon is attributed to the larger surface curvature at fruit edges, which causes distortion during the projection and reflection of structured light. After profile correction, the diffuse reflectance images show uniformly distributed overall brightness, with the intensity differences between edge and central regions effectively eliminated. This verifies the capability of the correction method to compensate for geometric distortion. Similarly, in the RGB images corresponding to optical transport coefficients, uncorrected images display spatial non-uniformity between peripheral and central regions, while corrected images present a homogeneous color distribution. This indicates the restoration of spatial uniformity in the optical transport coefficient maps.

The correction performance was further quantified using the CV value, which reflects the uniformity of optical properties across the fruit surface. A smaller CV value denotes a more concentrated diffuse reflectance distribution and higher optical uniformity on the fruit surface. Quantitative analysis revealed that the mean CV values of apples and pears before correction were 0.162 and 0.194, respectively, and these values decreased to 0.093 and 0.113 after correction. These results confirm that the surface contour correction technique effectively mitigates the impacts of non-planar surface geometry, yielding a more uniform diffuse reflectance distribution. Meanwhile, it achieves good compatibility with the planar characteristics of the simulation dataset.

### 3.2. Comparison and Analysis of Optical Transport Coefficient Prediction Performance

#### 3.2.1. Training Process Visualization and Analysis

**Figure 5:**
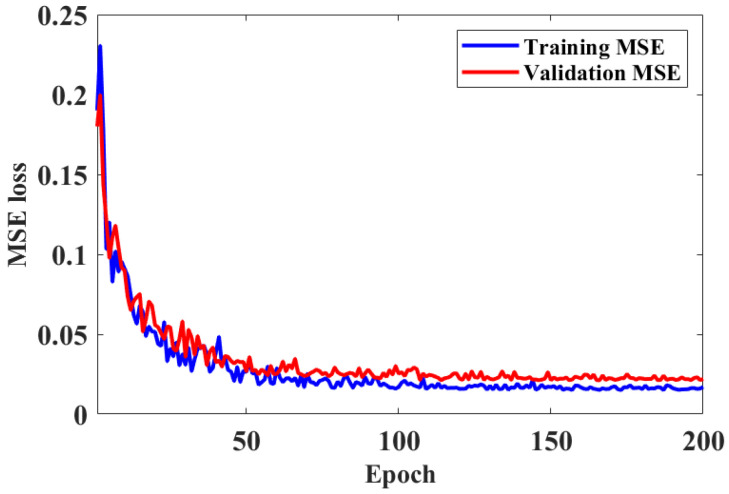
MSE loss plot of training set and validation set.

To verify the stability and convergence of the proposed CBAM-GAN-U-Net model during training, the model was iteratively trained on the simulation dataset. The variations in mean squared error (MSE) loss for both the training and validation sets were recorded for analysis (Figure 5). As shown in the figure, the MSE loss declined rapidly in the early stage of training and gradually stabilized after approximately 100 epochs. Throughout the entire training process, the loss gap between the training and validation sets remained small, indicating the absence of overfitting or underfitting issues. These results confirm that the training process was stable and well-converged, thereby providing a reliable foundation for the subsequent prediction of optical transport coefficients.

#### 3.2.2. Liquid Phantom Experiments and Validation

**Figure 6:**
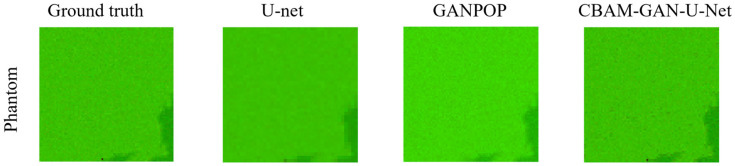
Prediction performance of different deep learning networks on phantom RGB optical coefficient maps.

Figure 6 illustrates the performance differences in the three models in predicting the optical coefficients of liquid phantoms. U-Net yields predictions with a typical checkerboard artifact, where the optical coefficient distributions are discretized and unable to form smooth, continuous mappings. This phenomenon is mainly attributed to the inherent architectural limitations of U-Net, which relies on a symmetric encoder–decoder structure and lacks an effective mechanism for global information integration. In contrast, both GANPOP and CBAM-GAN-U-Net can effectively suppress such checkerboard artifacts and significantly enhance the continuity of optical coefficient distributions.

The quantitative metrics presented in Table 1 further corroborate the visual observations. Clear performance hierarchies are observed among the three models, with each model demonstrating consistent prediction performance for both types of optical coefficients. Overall, CBAM-GAN-U-Net achieves the lowest NMAE, slightly outperforming GANPOP and substantially outperforming U-Net. This performance ranking can be attributed to inherent architectural differences between the models: U-Net lacks adversarial training and attention mechanisms, which restricts its ability to jointly capture global and local features. GANPOP enhances the realism of optical coefficient prediction through generator–discriminator adversarial learning, yet has insufficient capacity for effective feature selection. The CBAM-GAN-U-Net addresses this limitation by integrating attention modules, thus realizing simultaneous improvements in feature extraction efficiency and prediction accuracy. It is noteworthy that all three models exhibit slightly but consistently lower prediction accuracy for the absorption coefficient *μ_a_* than for the reduced scattering coefficient *μ_s_*′. As liquid phantoms are ideal samples free from texture interference, these results validate the rationality of the CBAM-GAN-U-Net architectural design and provide a baseline performance reference for subsequent optical coefficient prediction on real fruit samples.

#### 3.2.3. Apple Experiments and Performance Evaluation

Optical transport coefficients of non-bruised and bruised apples were measured using conventional SFDI. The mean absorption coefficient of non-bruised apples was 0.0324 mm^−1^, and the mean reduced scattering coefficient was 1.726 mm^−1^. Under bruised conditions, the mean absorption coefficient changed only marginally to 0.0331 mm^−1^, whereas the mean reduced scattering coefficient decreased significantly to 1.215 mm^−1^. This trend confirms that the reduced scattering coefficient is more sensitive to bruise-induced tissue structural damage, while the absorption coefficient is only weakly affected, in agreement with previous findings.

**Figure 7:**
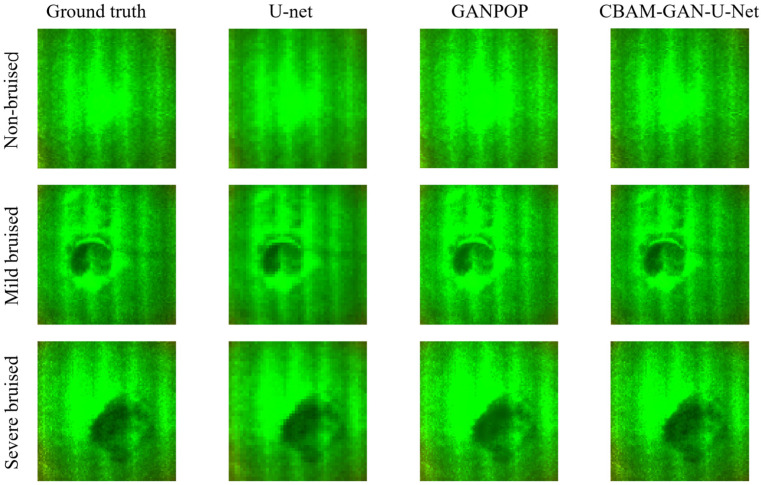
Prediction performance of different deep learning networks on the RGB optical coefficient map of apples.

Figure 7 presents the prediction results of the three models on apple samples. Due to the smooth surface and minimal texture interference of apples, these samples more clearly highlight the models’ ability to capture intrinsic optical characteristics of real fruits. The U-Net predictions still exhibit pronounced checkerboard artifacts, and as bruise severity increases, these artifacts overlap with bruise features, resulting in blurred bruise boundaries. Although GANPOP eliminates checkerboard artifacts, it fails to capture subtle bruise-related variations, indicating limited sensitivity to weak features. In contrast, CBAM-GAN-U-Net demonstrates superior feature reconstruction capability: the coefficient distributions of non-bruised samples remain stable, and the boundaries of mildly and severely bruised regions are clearly delineated. This performance is attributed to the adaptive focusing capability of the CBAM module on weak but relevant features.

Clear trends are observed from the metrics summarized in Table 2: for all three models, prediction errors rise and similarity metrics decline as bruise severity increases, while the extent of performance degradation varies substantially across the models. U-Net undergoes the most severe performance degradation, which reflects its limited adaptability to escalating feature complexity and inability to address bruise-induced optical variations. GANPOP experiences a milder degree of degradation by comparison. CBAM-GAN-U-Net shows the smallest performance degradation with increasing bruise severity and maintains high prediction accuracy, demonstrating robust adaptability to complex feature distributions. This performance disparity can be attributed to the inherent differences in the feature extraction strategies of the three models: U-Net lacks an effective feature selection mechanism, rendering it susceptible to an increase in feature complexity. GANPOP leverages adversarial training to enhance prediction performance, yet remains limited in capturing weak bruise-related features. CBAM-GAN-U-Net, via its integrated attention modules, adaptively focuses on bruise-related features while suppressing irrelevant background information, thereby enabling the accurate extraction of key optical characteristics even with increasing bruise severity.

#### 3.2.4. Pear Experiments and Performance Evaluation

Optical transport coefficients of non-bruised and bruised pears were also determined via conventional SFDI. For non-bruised pears, the mean absorption coefficient was 0.0418 mm^−1,^ and the mean reduced scattering coefficient was 2.102 mm^−1^. For bruised pears, the mean absorption coefficient exhibited only a minor variation to 0.0424 mm^−1^, while the mean reduced scattering coefficient decreased significantly to 1.702 mm^−1^—this trend again verifies the high sensitivity of the reduced scattering coefficient to bruise-induced tissue damage. Pears exhibited a generally higher reduced scattering coefficient than apples across both intact and bruised states.

**Figure 8:**
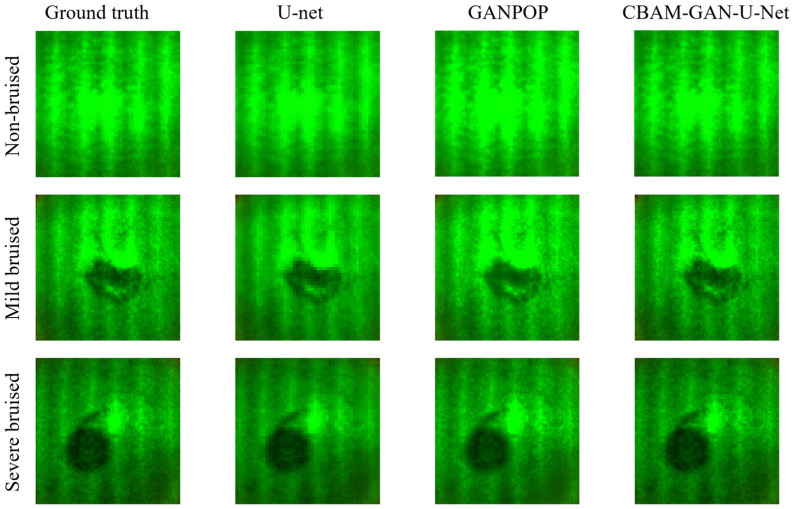
Prediction performance of different deep learning networks on the RGB optical coefficient map of pears.

Figure 8 presents the optical coefficient prediction results of the three models for pear samples. Pear surfaces contain lenticels and sporadic brown spots, leading to significantly more pronounced optical interference than that on apple surfaces, which places higher demands on the models’ robustness to surface texture interference. U-Net yields predictions severely affected by surface texture interference, reflecting its poor resistance to complex surface disturbances. GANPOP can suppress partial texture-related interference yet still produces minor prediction distortions in bruise regions. In contrast, the CBAM-GAN-U-Net generates clear and distinguishable optical coefficient distributions in bruise regions, and accurately reconstructs both the subtle optical variations associated with mild bruising and the spatial scope of severe bruising. These results visually validate the core advantages of the CBAM module in adaptive channel selection and targeted spatial focusing for bruise-related feature extraction.

The quantitative results presented in Table 3 clearly demonstrate that pear samples pose a greater challenge for optical coefficient prediction than apple samples. Nonetheless, the performance hierarchy of the three models remains consistent, with CBAM-GAN-U-Net outperforming GANPOP, which in turn surpasses U-Net. U-Net yields the poorest performance on pear samples, exhibiting significantly higher prediction errors even for non-bruised specimens compared with apple samples. GANPOP demonstrates markedly enhanced robustness to optical interference with a substantial reduction in prediction errors, yet its prediction accuracy remains constrained due to the absence of a targeted feature selection mechanism. In contrast, CBAM-GAN-U-Net consistently maintains good, stable prediction performance, achieving high accuracy even for pear samples with severe bruising. The prediction results for both pear and apple samples further verify that the architectural advantages of CBAM-GAN-U-Net are transferable across different fruit species.

Statistical test results showed that the NMAE of the proposed CBAM-GAN-U-Net model was significantly lower than that of the two baseline models on all test sample sets. Specifically, compared with the U-Net model, the prediction error reduction in our model reached an ultra-significant level on liquid phantoms, green apples and crown pears (all *p* < 0.001); compared with the GANPOP model, the performance improvement of our model also reached an extremely significant level on all three sample sets (all *p* < 0.01). These results confirm that the performance superiority of the proposed model is not caused by random data fluctuation, but has stable statistical significance.

The above results demonstrate that the simulation-trained CBAM-GAN-U-Net model achieves high-precision prediction of the reduced scattering coefficient *μ_s_*′ on independent real liquid phantoms, green apples and crown pears. This high prediction accuracy of *μ_s_*′, the core indicator for bruise discrimination, lays a reliable foundation for the subsequent subsurface fruit bruise discrimination experiments.

#### 3.2.5. Inference Efficiency and Real-Time Capability Analysis

The problem of conventional SFDI technology is the extremely low efficiency of the nonlinear least squares (NLS) optical property inversion process, which severely limits the real-time application of the method. To quantitatively verify the real-time performance, computational cost, and hardware adaptability of the proposed CBAM-GAN-U-Net model, we conducted an inference speed comparison between the proposed model and the conventional NLS inversion method, and evaluated its GPU resource consumption for both training and inference stages under a unified hardware and software environment.

All tests were completed in a unified environment with the following configuration: hardware includes an NVIDIA GeForce RTX 4060 Laptop GPU (8 GB VRAM), and 16 GB of RAM; the software environment is PyTorch 2.2.0 with CUDA 12.1. All tests used a 256 × 256-pixel SFDI diffuse reflectance image as the unified input, and the NLS inversion adopted the same convergence threshold and iteration settings consistent with conventional SFDI workflows in published studies to ensure the fairness of the comparison.

Quantitative comparison results show that the conventional NLS inversion method has an average processing time of 11.68 min (700.8 s) per single image, with a corresponding frame rate of only 0.0014 FPS, which cannot meet the basic requirements of real-time processing. In contrast, the proposed CBAM-GAN-U-Net model achieves an average inference time of 183 ms (0.183 s) per image, with a corresponding frame rate of 5.46 FPS, realizing a ≈3830× speedup over the conventional method. For GPU resource consumption, the model can be fully trained on the aforementioned RTX 4060 Laptop GPU within 4.2 h, with a peak VRAM usage of 7.2 GB during training; during inference, the model has a peak VRAM occupancy of only 1.4 GB. These results fully validate the excellent real-time processing capability and low deployment threshold of our proposed method.

### 3.3. Fruit Bruise Discrimination Results

Based on the high-precision *μ_s_*′ prediction results for real fruit samples above, this section evaluates the subsurface bruise discrimination performance of the proposed method. As fruit bruising causes significant changes in the reduced scattering coefficient *μ_s_*′ (the core indicator for discrimination), the high prediction accuracy of *μ_s_*′ on real samples ensures that the spatial distribution difference between sound and bruised tissues can be accurately captured, which is the prerequisite for reliable bruise discrimination. All discrimination tests in this section are performed on real green apple and crown pear samples, using the *μ_s_*′ maps predicted from actual SFDI measurement data, with the real bruise state of the samples as the standard for accuracy calculation.

#### 3.3.1. Discrimination Between Bruised and Non-Bruised Fruits

**Figure 9:**
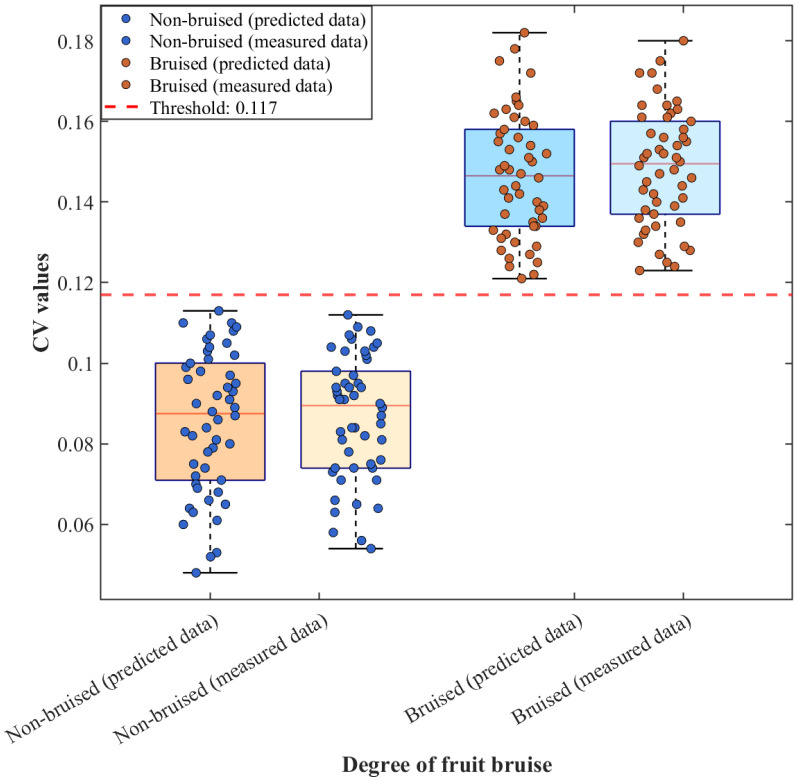
Box plot of CV values for apple predicted data and SFDI measured data.

Figure 9 presents box–scatter plots of the coefficient of variation (CV) of the reduced scattering coefficient *μ_s_*′ for non-bruised and bruised apple samples, derived from both predicted data and SFDI-measured data. The CV distributions of the two sample categories exhibit clear separation with no overlap. The CV ranges obtained from predicted data for non-bruised apples are highly consistent with those obtained from SFDI measurements and are concentrated within a low-value range, indicating uniform flesh tissue structure and stable *μ_s_*′ distributions in sound apples. Similarly, the CV distributions of bruised apples derived from both data sources remain consistent and are significantly higher than those of non-bruised samples. This increase is attributed to cell rupture and enhanced structural heterogeneity induced by bruising, which markedly increases the dispersion of the *μ_s_*′ distribution. Using a threshold of 0.117, both CV values derived from predicted data and those obtained directly from SFDI measurements achieved 100% classification accuracy for non-bruised versus bruised apples. These results not only confirm the strong discriminative capability of the CV metric for apple bruise status but also demonstrate the high consistency between the *μ_s_*′ values predicted by the CBAM-GAN-U-Net model and those measured by SFDI. CV-based discrimination using predicted data and measured data both effectively distinguish non-bruised and bruised apples.

**Figure 10:**
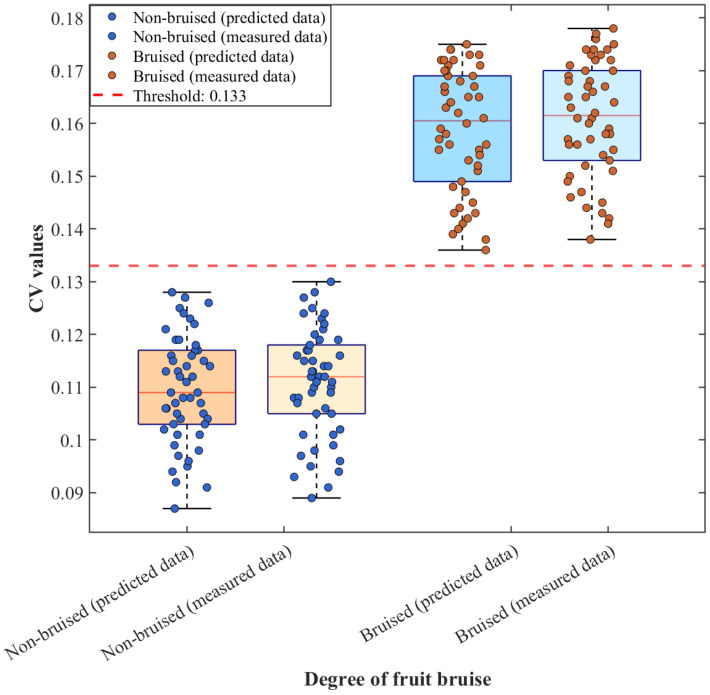
Box plot of CV values for pear predicted data and SFDI measured data.

Figure 10 shows the CV box–scatter plots for pear samples based on the two data sources. Despite optical interference from surface lenticels and sporadic brown spots on pears, the CV distributions still exhibit clear classification boundaries. With a threshold of 0.133, the discrimination accuracy for non-bruised versus bruised pears also reached 100%. From the distribution characteristics, the predicted and measured CV values for non-bruised pears show substantial overlap and stable concentration, whereas the CV values for bruised pears are significantly higher than those of non-bruised samples. The overall distribution trends of predicted and measured data are highly consistent, with only minor differences in dispersion, which can be attributed to slight surface interference affecting the measured data but not disrupting the classification boundary. These results indicate that CV values derived from predicted data provide discriminative performance comparable to that obtained from SFDI measurements, demonstrating the reliability of the simulation-driven prediction data. Even for samples with more complex surface structures, CV-based discrimination using predicted data maintains robust performance, further highlighting the generalizability and robustness of the proposed approach.

#### 3.3.2. Discrimination Between Mild Bruised and Severe Bruised Fruits

**Figure 11:**
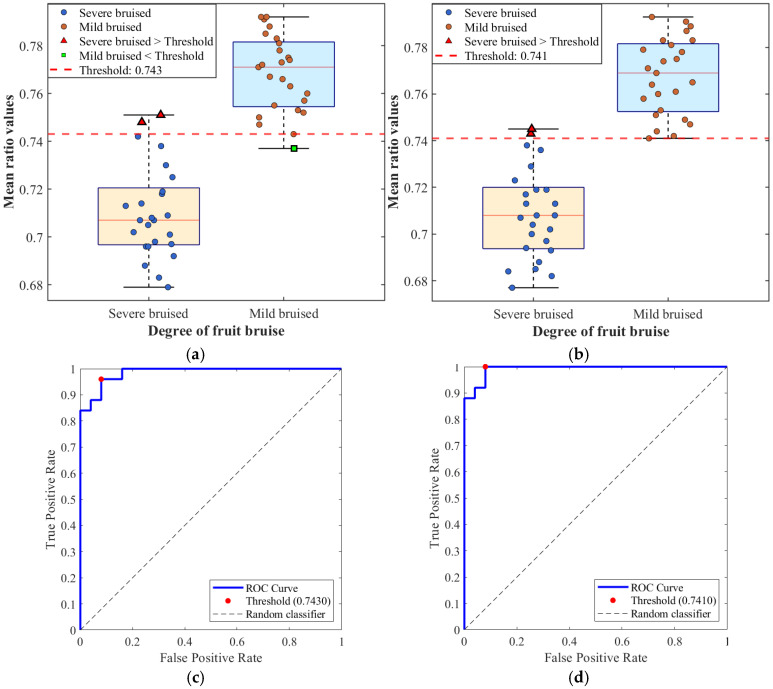
Results of bruise discrimination in apples: Box plots of mean ratio values for (**a**) predicted data and (**b**) SFDI measured data; ROC curves of mean ratio values for (**c**) predicted data and (**d**) SFDI measured data.

Figure 11 and Table 4 present the results of apple bruise severity discrimination based on the mean ratio derived from predicted data and SFDI-measured data. Both data sources exhibit similarly strong performance, primarily due to the ability of the mean ratio to capture tissue structural differences induced by bruising. From the distribution characteristics, mildly bruised apples exhibit relatively small and shallow cellular damage, leading to a gradual decrease in the mean *μ_s_*′ and correspondingly higher mean ratio values. In contrast, severe bruising causes extensive cell rupture and pronounced structural damage, resulting in a substantial decrease in the mean *μ_s_*′ and mean ratio values concentrated in a lower range. Although a slight overlap exists between the two classes, ROC-based threshold optimization ensures robust discrimination performance. For measured data, the AUC reaches 0.9920 with a sensitivity of 100%, indicating that all severely bruised samples are correctly identified. For predicted data, the AUC is 0.9856, with sensitivity and specificity of 96.00% and 92.00%, respectively, showing only minor differences. The optimal thresholds derived from both data sources are very close, reflecting the high consistency in distribution characteristics between the *μ_s_*′ values predicted by the CBAM-GAN-U-Net model and those measured by SFDI. In terms of final classification results, the overall misclassification rate is only 6% for predicted data and 4% for measured data, indicating that the model trained on simulation data produces outputs that closely approximate conventional SFDI measurements, thereby ensuring reliable discrimination accuracy.

**Figure 12:**
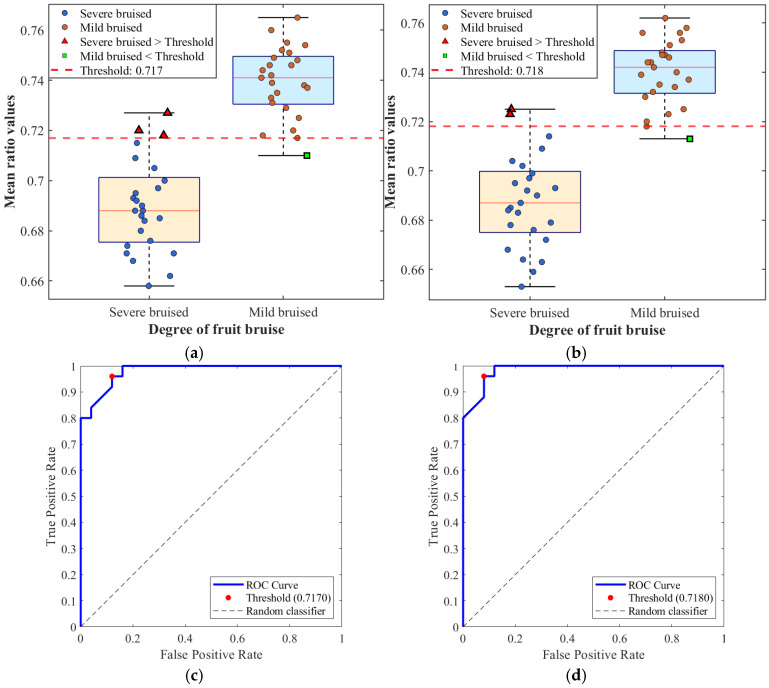
Results of bruise discrimination in pears: Box plots of mean ratio values for (**a**) predicted data and (**b**) SFDI measured data; ROC curves of mean ratio values for (**c**) predicted data and (**d**) SFDI measured data.

Figure 12 and Table 5 present the pear bruise severity discrimination results based on mean ratio values derived from predicted and measured data. Although pear bruise discrimination is affected by optical interference from surface lenticels and brown spots, the overall discrimination performance remains stable and reliable. Similar to apples, mildly bruised pears exhibit higher mean ratio values than severely bruised pears. However, due to the more dispersed spatial distribution of cellular damage in mildly bruised pears, the decrease in the mean *μ_s_*′ is more gradual, resulting in a smaller separation between mild and severe bruising compared with apples and slightly increased data overlap. Nevertheless, ROC analysis of both predicted and measured data still demonstrates strong discriminative capability. The AUC values for predicted and measured data are 0.9808 and 0.9856, respectively. And the optimal thresholds are highly similar, indicating that mean-ratio-based discrimination using predicted data closely matches that based on SFDI measurements. The final misclassification rates (Table 6) are 8% for predicted data and 6% for measured data. Although slightly higher than those for apples, these rates remain low, and the performance gap between the two data sources is minimal. This demonstrates that the proposed method maintains good adaptability to samples with more complex surface structures and stronger optical interference, highlighting the model’s robustness and generalization capability.

Considering the overall discrimination performance for both apples and pears, mean ratio values derived from predicted data and SFDI-measured data consistently exhibit highly similar discriminative effectiveness across different fruit types and surface structures, achieving accurate and stable bruise discrimination. The close agreement between predicted and measured data in terms of ROC metrics, optimal thresholds, and classification accuracy is fundamentally attributed to the accurate reproduction of optical transport physics in the Blender simulation environment and the effective capture of weak bruise-related features by the CBAM modules. Although pears exhibit slightly higher misclassification rates than apples due to more complex surface interference, this difference does not compromise overall discrimination accuracy and instead further corroborates the generalizability of the proposed approach.

## 4. Conclusions

To address the low efficiency of conventional inversion in SFDI and the scarcity of large-scale training datasets for deep learning, this study proposes a Blender simulation–driven deep learning framework for optical transport coefficient prediction. By integrating structured-light-based three-dimensional contour correction with an improved CBAM-GAN-U-Net architecture, a complete technical pipeline for subsurface fruit bruise detection and discrimination is established. The feasibility of the proposed approach is systematically validated through experiments on liquid phantoms, green apples, and crown pears.

An SFDI simulation environment was constructed in Blender, where the Cycles ray-tracing engine was used to reproduce the physical process of light transport. A total of 800 paired datasets of diffuse reflectance images and optical transport coefficient labels were generated in batch, effectively addressing the long preparation cycle and high cost associated with real dataset acquisition and providing abundant low-cost annotated samples for model training. The CBAM-GAN-U-Net model embeds CBAM into the GANPOP architecture, enabling dual optimization in channel and spatial dimensions. This significantly enhances the model’s ability to capture weak bruise-related features and resist surface texture interference. The NMAE of the reduced scattering coefficient prediction is as low as 0.021 ± 0.007 for liquid phantoms, 0.039 ± 0.012 for severely bruised green apples, and remains at 0.044 ± 0.015 for crown pears with complex surface structures, consistently outperforming U-Net and GANPOP models.

Based on the predicted reduced scattering coefficient *μ_s_*′, a discrimination strategy combining the coefficient of variation, mean ratio, and ROC curve analysis achieves efficient bruise classification. The CV metric attains 100% accuracy for non-bruised/bruised classification in both green apples and crown pears, while the mean ratio yields low misclassification rates of only 6% for green apples and 8% for crown pears in mild/severe bruise discrimination. In addition, the contour correction technique effectively eliminates geometric distortion caused by non-planar fruit surfaces, ensuring data compatibility and detection robustness.

In conclusion, this study proposes a Blender simulation-driven deep learning framework for fruit subsurface bruise detection, which solves the core problems of low efficiency of traditional SFDI inversion and the high cost and long cycle of real training dataset acquisition. On the basis of existing simulation research, this work makes targeted breakthroughs for key problems in fruit bruise detection. We construct a complete simulation-driven technical framework to tackle the pain point of real dataset acquisition. We adopt a profile correction method to bridge the simulation-to-real domain gap, boosting the model’s generalization performance on real fruit samples. We develop the CBAM-GAN-U-Net model with stronger weak bruise feature capture ability, which outperforms mainstream models in real sample optical coefficient prediction accuracy, and adopt a multi-index discrimination strategy based on the model-predicted optical coefficients, achieving 100% non-bruised/bruised classification accuracy and low misclassification rates for mild/severe bruise differentiation on real apples and pears. These form the core innovations of this study, and the proposed method realizes accurate and rapid subsurface bruise detection on real fruit samples.

From the perspective of practical agricultural production and application, the core practical conclusion of this study for fruit bruise non-destructive detection is that the proposed simulation-driven technical framework can achieve high-precision, low-cost non-destructive detection of hidden early subsurface bruises without relying on large-scale real labeled datasets. This method has significant agricultural application value. It can effectively reduce postharvest economic losses by identifying hidden bruises before storage and transportation, thus avoiding fruit deterioration and batch cross-contamination during circulation. It also improves the efficiency of postharvest fruit sorting compared with traditional manual inspection, which has important practical value for reducing postharvest losses in the fruit supply chain and provides a reliable technical solution for the fruit industry.

It should be noted that the proposed method is fully validated for subcutaneous bruise detection of green apples and crown pears under controlled laboratory conditions. For broader application scenarios, the performance of the method is constrained by the current experimental limitations, including the single-wavelength/single-spatial frequency parameter design, the optical parameter coverage of the simulation dataset, and the controlled experimental environment. The robustness and generalization ability of the method for other fruit varieties and high-speed sorting scenarios need to be further verified and optimized in future research.

## Figures and Tables

**Table 1:** Phantom image prediction quality metrics.

Deep Learning Network	Optical Transport Coefficient	NMAE	PSNR	SSIM
U-Net	*μ_s_*′	0.102 ± 0.018	31.5 ± 1.8	0.832 ± 0.025
	*μ_a_*	0.115 ± 0.020	30.2 ± 1.9	0.815 ± 0.028
GANPOP	*μ_s_*′	0.024 ± 0.011	38.8 ± 2.1	0.923 ± 0.018
	*μ_a_*	0.030 ± 0.012	37.5 ± 2.2	0.908 ± 0.020
CBAM-GAN-U-Net	*μ_s_*′	0.021 ± 0.007	45.2 ± 3.0	0.968 ± 0.011
	*μ_a_*	0.025 ± 0.008	43.1 ± 2.8	0.952 ± 0.013

**Table 2:** Apple image prediction quality metrics.

Deep Learning Networks	Bruise Level	Optical Transport Coefficient	NMAE	PSNR (dB)	SSIM
U-net	non-bruised	*μ_s_*′	0.128 ± 0.015	33.2 ± 2.0	0.856 ± 0.022
		*μ_a_*	0.142 ± 0.018	31.5 ± 2.1	0.830 ± 0.025
	mild bruised	*μ_s_*′	0.172 ± 0.020	29.8 ± 2.3	0.805 ± 0.028
		*μ_a_*	0.188 ± 0.023	28.0 ± 2.4	0.782 ± 0.030
	severe bruised	*μ_s_*′	0.215 ± 0.025	26.5 ± 2.5	0.763 ± 0.032
		*μ_a_*	0.235 ± 0.028	24.7 ± 2.6	0.738 ± 0.035
GANPOP	non-bruised	*μ_s_*′	0.042 ± 0.010	40.5 ± 2.4	0.931 ± 0.015
		*μ_a_*	0.050 ± 0.012	38.2 ± 2.5	0.905 ± 0.018
	mild bruised	*μ_s_*′	0.047 ± 0.012	35.6 ± 2.7	0.892 ± 0.020
		*μ_a_*	0.056 ± 0.014	33.5 ± 2.8	0.868 ± 0.022
	severe bruised	*μ_s_*′	0.052 ± 0.016	32.2 ± 3.0	0.858 ± 0.024
		*μ_a_*	0.063 ± 0.018	30.1 ± 3.1	0.832 ± 0.026
CBAM-GAN-U-Net	non-bruised	*μ_s_*′	0.023 ± 0.008	43.8 ± 2.6	0.958 ± 0.010
		*μ_a_*	0.030 ± 0.009	41.0 ± 2.7	0.932 ± 0.012
	mild bruised	*μ_s_*′	0.032 ± 0.011	39.5 ± 2.9	0.932 ± 0.014
		*μ_a_*	0.038 ± 0.012	37.2 ± 2.9	0.898 ± 0.015
	severe bruised	*μ_s_*′	0.039 ± 0.012	35.8 ± 3.2	0.905 ± 0.018
		*μ_a_*	0.047 ± 0.014	33.6 ± 3.3	0.870 ± 0.019

**Table 3:** Pear image prediction quality metrics.

Deep Learning Networks	Bruise Level	Optical Transport Coefficient	NMAE	PSNR (dB)	SSIM
U-net	non-bruised	*μ_s_*′	0.185 ± 0.022	28.6 ± 2.2	0.798 ± 0.030
		*μ_a_*	0.222 ± 0.025	27.0 ± 2.3	0.774 ± 0.032
	mild bruised	*μ_s_*′	0.223 ± 0.028	25.3 ± 2.4	0.745 ± 0.035
		*μ_a_*	0.268 ± 0.030	23.8 ± 2.5	0.723 ± 0.037
	severe bruised	*μ_s_*′	0.265 ± 0.032	22.8 ± 2.7	0.702 ± 0.040
		*μ_a_*	0.318 ± 0.035	21.4 ± 2.8	0.681 ± 0.042
GANPOP	non-bruised	*μ_s_*′	0.043 ± 0.012	34.8 ± 2.6	0.885 ± 0.022
		*μ_a_*	0.052 ± 0.014	32.7 ± 2.7	0.858 ± 0.024
	mild bruised	*μ_s_*′	0.051 ± 0.017	31.5 ± 2.9	0.836 ± 0.027
		*μ_a_*	0.061 ± 0.019	29.6 ± 3.0	0.811 ± 0.029
	severe bruised	*μ_s_*′	0.055 ± 0.019	28.6 ± 3.2	0.805 ± 0.030
		*μ_a_*	0.066 ± 0.021	26.9 ± 3.3	0.781 ± 0.032
CBAM-GAN-U-Net	non-bruised	*μ_s_*′	0.029 ± 0.010	39.2 ± 3.1	0.928 ± 0.016
		*μ_a_*	0.035 ± 0.011	36.8 ± 3.2	0.900 ± 0.017
	mild bruised	*μ_s_*′	0.035 ± 0.013	35.6 ± 3.3	0.892 ± 0.021
		*μ_a_*	0.042 ± 0.015	33.5 ± 3.4	0.865 ± 0.022
	severe bruised	*μ_s_*′	0.044 ± 0.015	32.4 ± 3.5	0.868 ± 0.025
		*μ_a_*	0.053 ± 0.017	30.5 ± 3.6	0.842 ± 0.026

**Table 4:** ROC analysis metrics for apple data.

Data Type	Optimal Threshold	AUC	Sensitivity	Specificity	Youden Index
predicted data	0.7430	0.9856	96.00%	92.00%	0.8800
measured data	0.7410	0.9920	100.00%	92.00%	0.9200

**Table 5:** ROC analysis metrics for pear data.

Data Type	Optimal Threshold	AUC	Sensitivity	Specificity	Youden Index
Predicted data	0.7170	0.9808	96.00%	88.00%	0.8400
Measured data	0.7180	0.9856	96.00%	92.00%	0.8800

**Table 6:** Discrimination results of mild and severe bruised fruits (based on Mean ratio).

Sample Type	Sample Grade	Actual Amount	Predicted Data (Fault/Rate)	SFDI Measured Data (Fault/Rate)
apples	mild bruised	25	1 4%	0 0%
	severe bruised	25	2 8%	2 8%
	Total	50	3 6%	2 4%
pears	mild bruised	25	1 4%	1 4%
	severe bruised	25	3 12%	2 8%
	total	50	4 8%	3 6%

## Data Availability

The original contributions presented in the study are included in the article, further inquiries can be directed to the corresponding author.
